# A case report of rib osteosarcoma and literature review

**DOI:** 10.1111/crj.13686

**Published:** 2023-09-14

**Authors:** Xiaofeng Hu, Tianyi Bao, Chao Yan, Yongliang Zhu, Xiaofei Zheng

**Affiliations:** ^1^ Department of Orthopaedics Jinling Hospital of Nanjing Medical University Nanjing China; ^2^ Department of Orthopaedics Nanjing Central Hospital Nanjing China

**Keywords:** osteosarcoma, rib, surgical treatment

## Abstract

About half of osteosarcomas occur near the knee joint, but other sites such as the humerus, upper femur, fibula, spine, and ilium can also occur. However, rib osteosarcoma is rarely reported. Here, we report the case of a 17‐year‐old female who was found to have a left dorsal mass on physical examination. Computed tomography (CT) revealed bone destruction in the seventh rib, leading to surgery for mass excision. Pathological results suggested chondroblastic osteosarcoma. After surgery, the patient was treated with chemotherapy and is doing well.

## Graphical abstract

Osteosarcoma of the ribs is very rare. Here, we report a case of primary osteosarcoma of the ribs in an adolescent girl. The patient underwent complete resection of the rib mass, and the mass was histopathologically diagnosed as chondroblastic osteosarcoma. After surgery, the patient received adjuvant chemotherapy and has no evidence of recurrence.

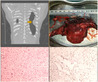

## 1 INTRODUCTION

Osteosarcoma is a malignant mesenchymal tumor. It is mainly manifested as the formation of immature osteoid cells by tumor cells. Osteosarcoma is the most common primary bone tumor in children and adolescents, with a tendency to occur in the metaphysis of the long shaft. Osteosarcoma occurring at the rib end is very rare, and here, we report a case of primary rib osteosarcoma treated in an adolescent girl in order to provide some diagnostic aid to clinicians.

## 2 CASE REPORT

**FIGURE 1:**
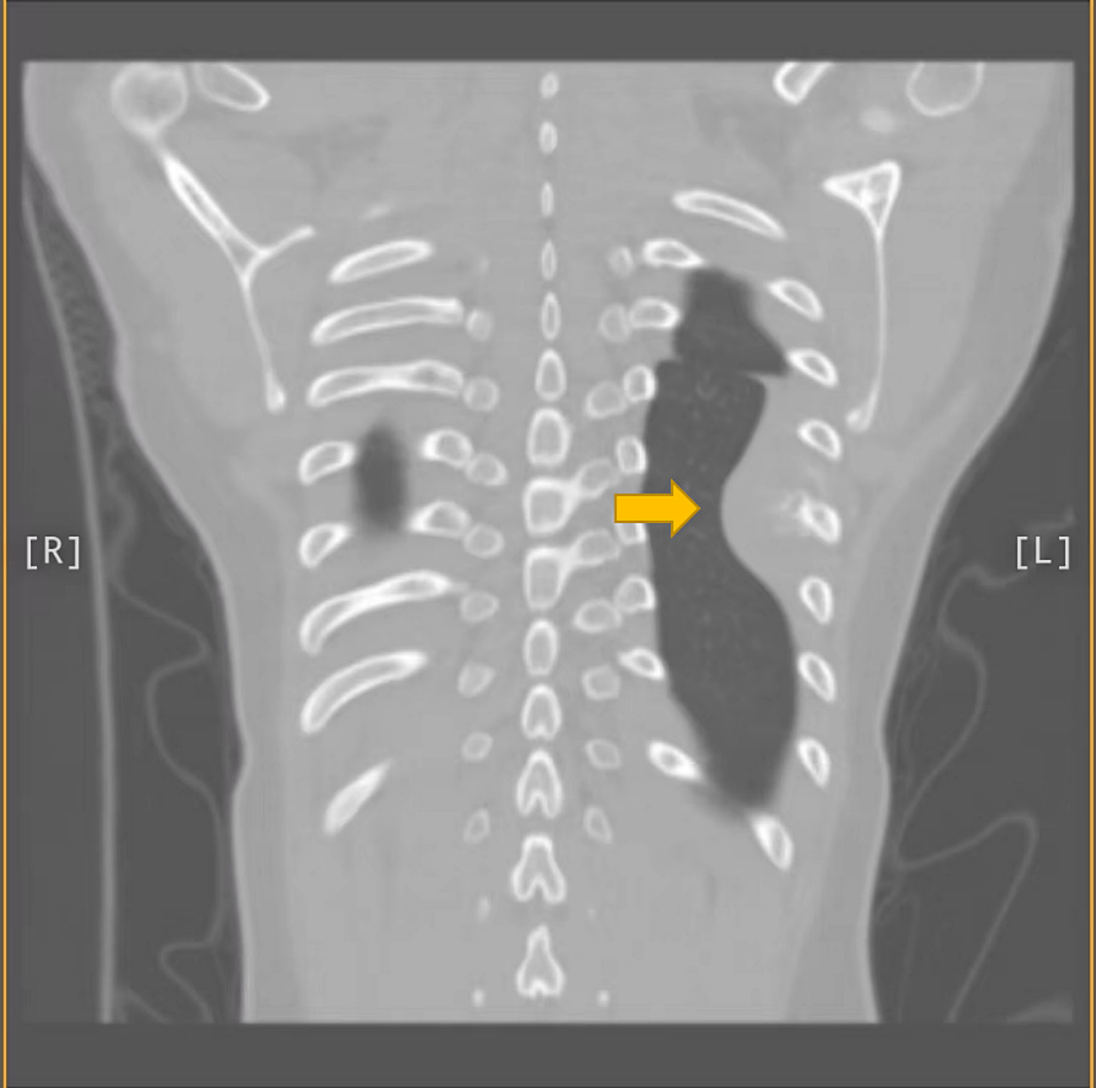
Preoperative coronal CT of patient. Notice a semicircle mass (pointed by yellow arrow) on left hemithorax and destruction of the seventh ribs.

**FIGURE 2:**
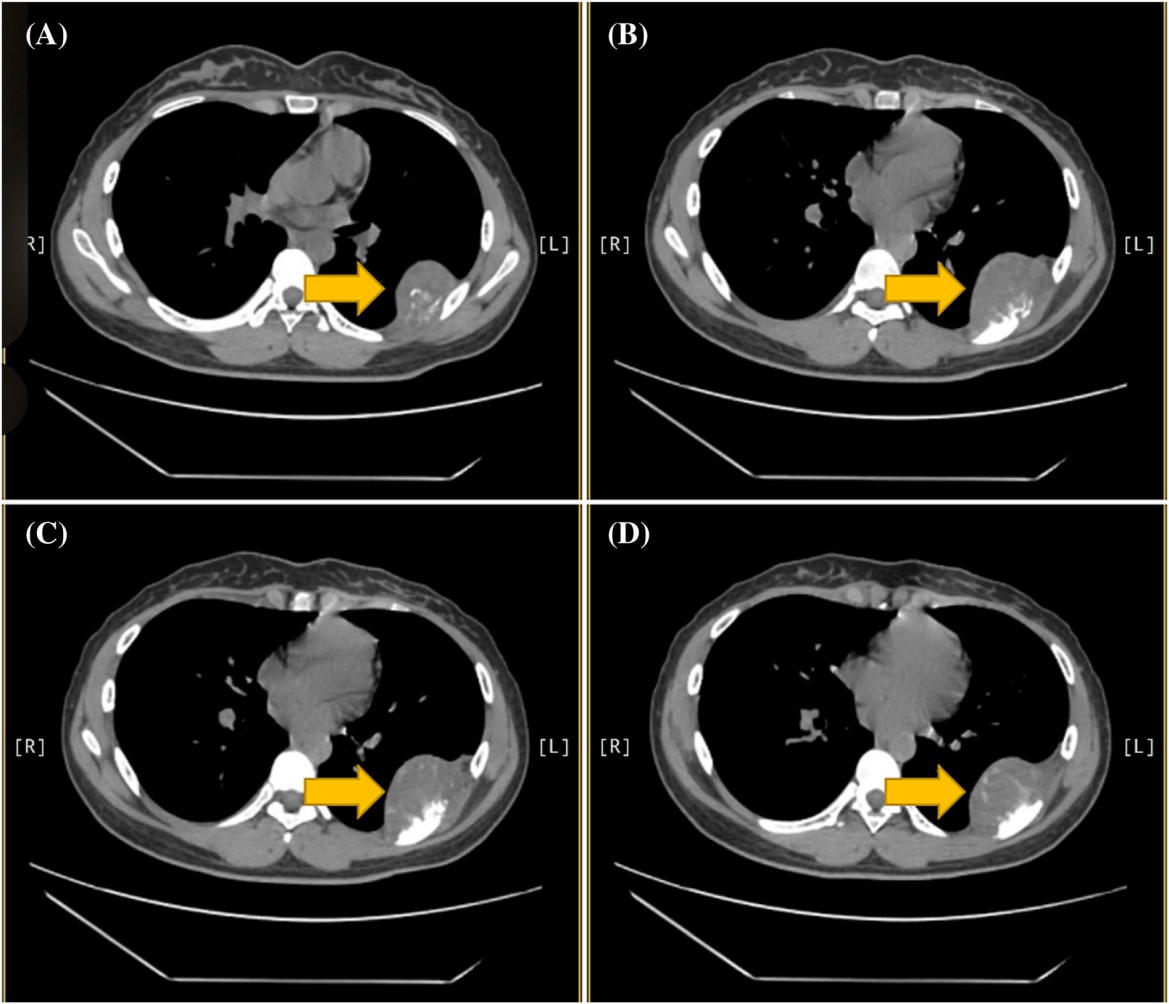
Preoperative CT showed destruction of the seventh rib and surrounded by mass (pointed by yellow arrow).

We admitted a 17‐year‐old adolescent girl. She has a 1‐month history of swelling and pain in her left chest and back. During a school‐organized physical examination 4 days ago, a lump was found on her left back. The patient has no symptoms such as fever, cough, dyspnea, and weight loss. We examined the patient and found that a lump could be felt at the level of approximately the seventh rib on the left chest and back, with an area of approximately 2.0 × 3.0 cm. The lump feels tough and lacks mobility, causing pain when pressed on this area. Complete blood cell count and serum biochemistry are within the normal range. Chest x‐ray showed rib destruction and surrounding mass. The results of chest computed tomography (CT) showed that the seventh rib on the left side of the patient was partially damaged, with a soft tissue mass surrounding it, and no pleural effusion was found (Figures 1 and 2). Emission computed tomography (ECT) showed that the local strip radioactive uptake of the left seventh posterior rib increased, indicating that bone metabolism was active.

**FIGURE 3:**
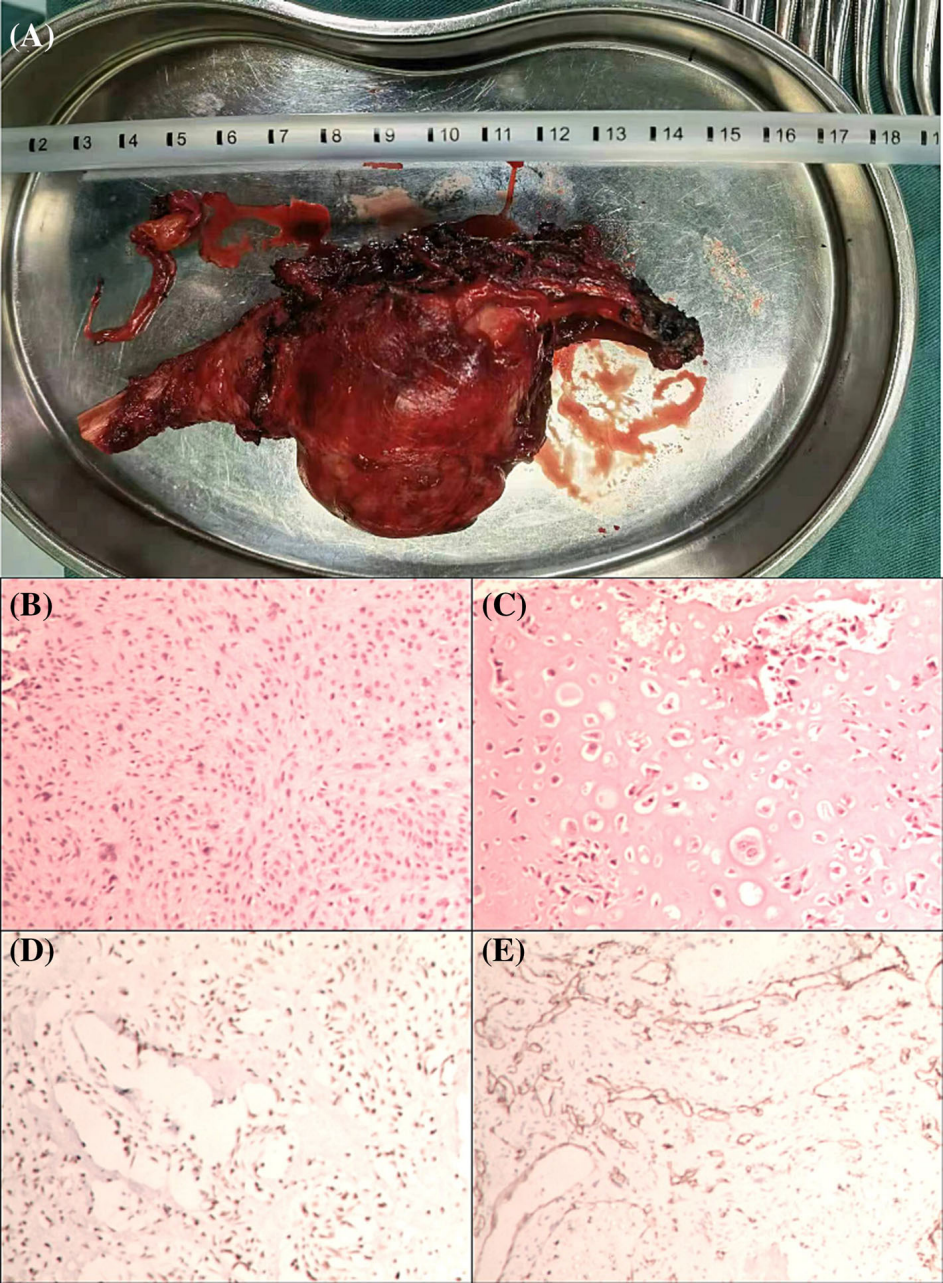
(A) Excised tumor mass, (B and C) HE staining picture of tissue, and (C and D) immunohistochemical staining pictures.

During the operation, the seventh rib mass protruded to the medial side. The mass is tough and has no obvious adhesion with the surrounding, and the capsule is intact. In order to completely remove the mass, we carefully separated the mass, removed part of the latissimus dorsi muscle, and cut the ribs at the normal tissue on both sides of the mass (Figure 3A). The mass was histopathologically diagnosed as chondroblastic osteosarcoma (Figure 3B,C). After surgery, the patient was transferred to the oncology department for chemotherapy. Chemotherapy was initiated with a protocol consisting of cyclophosphamide (2 g) in the first to third day, doxorubicin liposome (40 mg) in the first day and lobaplatin (40 mg) in the second day of the treatment. It is supplemented by symptomatic treatment such as stomach protection, liver protection, and antiemesis. After the treatment, the patient voluntarily asked for discharge. The patient is required to return to the hospital regularly for re‐examination.

The patient has lived normally for one and a half years after treatment, and no special changes have been found in the ribs or other parts of the body.

## 3 DISCUSSION

Osteosarcoma is one of the primary malignant tumors of the bone, which is more common in males. The incidence rate of osteosarcoma showed a bimodal pattern, reaching its peak at the age of 18 and 60 respectively.^1^ Osteosarcoma mainly occurs around the knee (50%) and near the humerus (15%).^2^ It is worth noting that osteosarcoma rarely occurs in flat bones, so there are few reported cases of rib osteosarcoma. Wardoyo et al. found that rib osteosarcoma accounts for only 1.25% of all osteosarcoma cases.^3^ Burt et al. reported 1435 cases of osteosarcoma, with only 13 cases (0.9%) of primary rib osteosarcoma.^4^ Similarly, Bielack et al. found 14 cases (0.8%) of rib osteosarcoma out of 1702 cases.^5^ Among all types of osteosarcoma patients, the incidence rate of male patients is about 1.5 times that of female patients.^6^

Almost all patients had lumps or pain or both, and some had other symptoms such as cough, dyspnea, and hemoptysis.^7^ The overall 5‐year survival rate for localized osteosarcoma is 60% to 80%,^6^ with a survival rate of approximately 60% for children and adolescents.^8^ Among metastatic osteosarcoma, patients with lung metastasis have the worst prognosis, with a 3‐year survival rate of less than 30%.^9^ Fractures may also be a more invasive indicator of diseases. Patients with fractures have a higher incidence of lung metastasis at the onset and after treatment.^10^

At present, the key population for screening osteosarcoma is mainly patients with hereditary cancer susceptibility syndrome. The screening strategy advocates increasing awareness of the risk of osteosarcoma and conducting comprehensive physical examinations every year. For patients carrying the pathogenic TP53 germline variants, it is necessary to focus on monitoring blood indicators and imaging changes, which can help physicians detect solid tumors in patients early and improve their long‐term survival rate.^11^ Recommendations for patients and families with Li‐Fraumeni syndrome include conducting annual whole‐body MRI examinations to screen for various possible malignant tumors, including sarcomas, and maintaining a high suspicion index for rare cancers.^12^

X‐ray examination is the standard examination for all patients with chest abnormalities. When examining patients with osteosarcoma, large lesions may be found, mainly manifested as destruction of normal bone trabeculae with unclear edges.^13^ Lesions usually stimulate the formation of new bone in the periosteum, resulting in the characteristic Codman triangle. For a more detailed evaluation, CT or magnetic resonance imaging (MRI) can be performed.^14^ CT scans can provide sufficiently detailed images to evaluate almost all chest wall tumors. MRI can better represent related soft tissue masses, which can provide effective information for subsequent biopsy and final surgical resection. MRI often captures jumping metastases of local tumors. But if it metastasizes further away, a full body bone scan is required, which has an impact on treatment and prognosis. According to the UICC‐TNM classification, 8 cm was defined as the prognostic cutoff for tumor size.^15^

The resection of primary osteosarcoma should be cautious to achieve complete resection while preserving bone function. Otherwise, intra lesion or marginal resection will increase the local recurrence rate, which is related to a decrease in overall survival rate.^5^, ^16^ The local recurrence rate of osteosarcoma in the limbs is relatively low, usually less than 5%, indicating that in most cases, complete resection can be achieved.^17^ Among patients with osteosarcoma who choose surgery, postoperative radiation therapy should be considered, especially for patients with closer surgical margins and lower degree of necrosis of the excised specimen.

In the English literature, we found about 17 cases of primary rib osteosarcomas. The characteristics of 18 patients with rib osteosarcoma, including this case, are shown in Table 1. As can be seen from the table, most patients can obtain a good therapeutic effect by surgery and adjuvant chemotherapy. The patient in this case was evaluated by experts in orthopedics and thoracic surgery immediately after the discovery of the chest mass. During the surgical resection of the mass, we removed at least 3 cm of normal rib tissue at both ends of the mass for complete removal of the mass. At the same time, the patient underwent chemotherapy after surgery. Our treatment strategy was broadly the same as the guidance given in the literature. The patient's postoperative feedback was also quite good, and no significant recurrence was found for one and a half years after the operation.

**TABLE 1:** The characteristics of the patients with osteosarcoma of the rib.

References	Age (years)	Sex	Location	Primary/secondary	Size (cm)	Pathology	Treatment	Outcome
Wardoyo et al.^3^	55	F	4th rib	Primary	5.5 × 5.3	Conventional osteosarcoma	Surgery	NA
Yaman Bajin et al.^7^	14	M	5th rib	Primary	10.0	Conventional osteosarcoma	CT	Died with disease, 12 months
Moghadamfalahi and Alatassi^18^	33	M	6th–8th ribs	Primary	4.0	Low‐grade central osteosarcoma	Surgery	NA
Bay et al.^19^	14	F	4th rib	Primary	12.0 × 13.0	Conventional osteosarcoma	NACT Surgery CT	Alive without disease, 8 months
Lim et al.^20^	15	M	4th rib	Primary	9.0 × 13.0	Conventional osteosarcoma	CT Surgery	Alive without disease, 10 months
Chattopadhyay et al.^21^	11	M	9th and 10th ribs	Primary	3.5 × 1.5	Conventional osteosarcoma	Surgery CT	Alive without disease, 24 months
Ikeda et al.^22^	37	F	2nd–4th ribs	Primary	8.0 × 7.0	High‐grade malignant osteosarcoma	Surgery CT	Alive without disease, 11 months
Botchu et al.^23^	7	F	7th rib	Primary	NA	Conventional osteosarcoma	Surgery ACT	Alive without disease, 12 months
Zheng et al.^24^	29	F	9th and 11th ribs	Primary	NA	Conventional osteosarcoma	CT Surgery Long‐term immunotherapy	Alive without disease, 60 months
Das et al.^25^	12	M	6th rib	Primary	7.0 × 3.5 × 2.5	Chondroblastic osteosarcoma	Surgery	NA
Sinn et al.^26^	29	F	3rd–5th ribs	Primary	NA	Conventional osteosarcoma	CT Surgery	Recurrence, 4.5 years
Xie and Huang^27^	23	M	8th–10th ribs	Primary	8.8 × 8.3	Conventional osteosarcoma	NA	NA
Krishnamurthy and Arulmolichelvan^28^	24	M	5th–7th ribs	Primary	20.0 × 15.0	High‐grade osteosarcoma	ACT Surgery CT	Alive without disease, 13 months
Hong et al.^29^	17	M	7th rib	Primary	5.0 × 6.0	Periosteal Osteosarcoma	Surgery	Alive without disease, 5 years
Xu and Zheng^30^	59	M	4th and 5th ribs	Primary	NA	Conventional osteosarcoma	Surgery	Recurrence, 1 month
Kuwabara et al.^31^	67	M	3rd and 4th ribs	Primary	12	Conventional osteosarcoma	Surgery	Died with disease, 4 months
Anoop et al.^32^	16	M	2nd rib	Primary	12.3 × 9.5 × 9.0	Conventional osteosarcoma	CT	Died with disease, 1 week
Our patient	17	F	7th rib	Primary	2.0 × 3.0	Chondroblastic osteosarcoma	Surgery CT	Alive without disease, 18 months

Abbreviations: ACT, adjuvant chemotherapy; CT, chemotherapy; F, female; M, male; NA, not available; NACT, neoadjuvant chemotherapy.

Several points should be noted in this case. This patient was found during physical examination, which is relatively early, and the surgical stage belongs to stage IIA (G2T1M0). According to our domestic treatment plan, surgery can be performed before chemotherapy. For stage IIB and III patients, neoadjuvant chemotherapy followed by surgery, and then chemotherapy. Preoperative neoadjuvant chemotherapy is very important for patients with limb salvage, but this patient's osteosarcoma is located in the ribs, and there is no need to reconstruct the bone structure. With or without preoperative chemotherapy, complete resection can be achieved without affecting the function. For patients with stage IIA rib osteosarcoma, early resection of the tumor also has certain advantages. For example, patients have good immunity and quick recovery after surgery. In addition, it can avoid some adverse consequences caused by puncture and obtain reliable pathological results at an early stage.

## AUTHOR CONTRIBUTIONS

**Xiaofeng Hu**: Study concept or design; data collection. **Tianyi Bao**: Data analysis or interpretation; writing the paper. **Chao Yan**: data collection. **Yongliang Zhu**: Data analysis or interpretation. **Xiaofei Zheng**: Study concept or design; writing the paper.

## CONFLICT OF INTEREST STATEMENT

The authors declare that they have no conflicts of interest.

## ETHICS STATEMENT

Appropriate written informed consent was obtained from the patient for the publication of this case report and accompanying images. It was approved by the Clinical Research Ethics Committee of Jinling Hospital of Nanjing Medical University and was implemented.

## Data Availability

Data sharing is not applicable to this article as no datasets were generated or analyzed during the current study.
